# A pharmacophore‐based classification better predicts the outcomes of HER2‐negative breast cancer patients receiving the anthracycline‐ and/or taxane‐based neoadjuvant chemotherapy

**DOI:** 10.1002/cam4.4022

**Published:** 2021-06-02

**Authors:** Xuan Li, Hefen Sun, Qiqi Liu, Yang Liu, Yifeng Hou, Wei Jin

**Affiliations:** ^1^ Department of Breast Surgery Key Laboratory of Breast Cancer in Shanghai Fudan University Shanghai Cancer Center Shanghai China; ^2^ Department of Oncology Shanghai Medical College Fudan University Shanghai China

**Keywords:** gene expression, HER2‐negative breast cancer, neoadjuvant chemotherapy, pharmacophore, prognosis

## Abstract

**Aims:**

Prognosis of patients for human epidermal growth factor receptor 2 (HER2)‐negative breast cancer post neoadjuvant chemotherapy is not well understood. The aim of this study was to develop a novel pharmacophore‐based signature to better classify and predict the risk of HER2‐negative patients after anthracycline‐and/or taxane‐based neoadjuvant chemotherapy (NACT).

**Main methods:**

Anthracycline and taxane pharmacophore‐based genes were obtained from PharmMapper. Drug‐targeted genes (DTG) related clinical and bioinformatic analyses were undertaken in four GEO datasets.

**Key findings:**

We used 12 genes from the pharmacophore to develop a DTG score (DTG‐S). The DTG‐S classification exhibited significant prognostic ability with respect to disease free survival (DFS) for HER2‐negative patients who receive at least one type of neoadjuvant chemotherapy that included anthracycline and/or taxane. DTG‐S associated with a high predictive ability for pathological complete response (pCR) as well as for prognosis of breast cancer. Using the DTG‐S classification in other prediction models may improve the reclassification accuracy for DFS. Combining the DTG‐S with other clinicopathological factors may further improve its predictive ability of patients’ outcomes. Gene ontology and KEGG pathway analysis showed that the biological processes of DTG‐S high group were associated with the cell cycle, cell migration, and cell signal transduction pathways. Targeted drug analysis shows that some CDK inhibitors and PI3K‐AKT pathway inhibitors may be useful for high DTG‐S patients.

**Significance:**

The DTG‐S classification adds prognostic and predictive information to classical parameters for HER2‐negative patients who receive anthracycline‐and/or taxane‐based NACT, which could improve the patients’ risk stratification and may help guide adjuvant treatment.

## Graphical abstract

Our pharmacophore‐based 12‐gene drug‐targeted genes score (DTG‐S) displays better predictive values for DFS and for pathological response to anthracycline‐and/or taxane‐based chemotherapy in human epidermal growth factor receptor 2 negative breast cancer. The biological relevance of the signature were cell cycle and cell signal transduction. The targeted drug analysis shows that some CDK inhibitors which can block the cell cycle and PI3K‐AKT pathway inhibitors may useful for high DTG‐S patients.

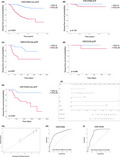

## 1 INTRODUCTION

Neoadjuvant chemotherapy (NACT) is often used for breast cancer patients to increase the operability for those with locally advanced breast tumors or to shrink the tumor size to enhance the possibility of breast‐conserving surgery for those with relatively large tumors.^1^ Another advantage of NACT is that it can reveal the chemosensitivity of patients, as a pathological complete response (pCR) after NACT is significantly associated with a prolonged relapse‐free survival and overall survival.^2^, ^3^, ^4^, ^5^ However, only a small percentage of patients achieve a pCR after NACT at a rate ranging from 10% to 50%.^6^, ^7^ This means that a large number of patients can be classified into a poor prognosis group after NACT, who could potentially recommend additional adjuvant treatment. The different rates in achieving a pCR can be partially explained by the heterogeneity of breast cancer, as the patient's tumor stage, histologic grade, age, hormonal status (estrogen receptor [ER], progesterone receptor [PR]), and human epidermal growth factor receptor 2 (HER2) status, as clinicopathological characteristics are closely associated with the probability of achieving a pCR.^8^ In recent years, thanks to the development of in‐depth sequencing technology in genomics, a large number of patients’ gene transcript expression data were made available to researchers. Many attempts have been made by using gene transcript expression data alone or in combination with other classical predictors to better predict the outcomes of patients who received NACT.

Multigene signatures such as Oncotype DX Recurrence Score,^9^ Mammaprint,^10^ risk of recurrence (ROR) scores^11^ and Gene Expression Grade Index (GGI) signature^12^ have been attracting increasing attention as they have been reported to improve the predictive ability for an individual patient's prognosis. These signatures can predict the distant recurrence of breast cancer in patients, and some also show an association with the probability of achieving a pCR. However, evidence of these signatures for use in NACT patients is limited and equivocal due to their design to target specific patients, such as the 21‐gene OncotypeDX recurrence score and the ROR score, which were designed for early‐stage invasive breast cancer patients with ER positive/HER2 negative type to guide decisions about systemic adjuvant treatment after surgical resection.^13^, ^14^, ^15^, ^16^, ^17^ Some of these predictors are reported to be associated with the sensitivity of breast cancer to NACT, such as the Diagonal Linear Discriminant Analysis‐30 genes (DLDA‐30) predictor and the Genomic Grade Index (GGI) predictor, both of which have been used in patients who received preoperative chemotherapy with paclitaxel, fluorouracil, doxorubicin, and cyclophosphamide, which identifies more pCR patients, but their prognostic value remains unclear.^12^, ^18^ Therefore, it is important to identify additional predictors that can determine the prognosis after NACT in breast cancer.

The National Comprehensive Cancer Network (NCCN) recommends that the chemotherapy regimens at best contain anthracycline and/or taxane for HER2‐negative breast cancer. There is a significant clinical need for a method that can identify patients who would benefit most from these treatments to avoid further adjuvant chemotherapy, which has many dose‐related side effects. Pharmacogenomics is studying the role of genetics in drug responses. It allows physicians to take into consideration their patients’ genetic makeup to make the treatment more “personalized”.^19^ A gene's relationship with pharmaceuticals may also provide some information about the treatment response and the patient prognosis. PharmMapper is an open‐source web server that can identify potential drug‐target pharmacophores, which may provide useful insights into bioassays for drug‐target research.^20^ In this study, we used anthracycline and taxane pharmacophore‐targeted genes to establish a risk score associated with the prognosis in HER2‐negative breast cancer patients who received at least one type of anthracycline‐ and/or taxane‐based NACT. We also explored the correlation of this risk score with clinicopathological variables and the pCR to neoadjuvant chemotherapy.

## 2 MATERIALS AND METHODS

### 2.1 Drug targeted genes (DTG) collection

The 3D structures of doxorubicin, epirubicin, and taxane were downloaded from PubChem (https://pubchem.ncbi.nlm.nih.gov/), and drug‐targeted genes (DTGs) and proteins were obtained from PharmMapper and ranked by calculating the PharmMapper fit score of the drug ligands using the drug's structure.^20^, ^21^ Homo sapiens genes with fit scores higher than 0.4 were considered DTGs.

### 2.2 RNA‐seq and clinical data collection

Clinical data and gene expression matrixes for HER2‐negative patients were recorded and downloaded from several Gene Expression Omnibus (GEO) datasets (https://www.ncbi.nlm.nih.gov/gds/). Eligibility standards were as follows: (1) breast cancer patients; (2) all patients received anthracycline‐based, taxane‐based or taxane‐anthracycline‐based neoadjuvant chemotherapy; and (3) the data had the pCR or survival information.

Eleven GEO datasets were included in our study. The discovery set was GSE25066 (treated with taxane‐anthracycline neoadjuvant chemotherapy) for pCR and DFS analysis (*N *= 466). The verification set included 10 independent GEO sets for different purposes (Table S1). Gene expression data from all of these datasets were magnitude normalized and log_2_ calculated.

### 2.3 Construction of the DTG‐S

The LASSO Cox regression model analysis was performed in R software (version 3.3.1) using the ‘glmnet’ package, and the DTGs with non‐zero coefficients were selected for further analysis. Weighted gene coexpression network analysis (WGCNA) was performed using the WGCNA module in R software. The appropriate soft threshold power β was 4, and the minimal module size was 15 in our study. Receiver operating curve (ROC) analysis and the area under the ROC (AUC) were used to confirm the predictive ability of the parameters for DFS or for pCR. The overlapping DTGs selected from the LASSO analysis and WGCNA were chosen as targeted genes. The DTG‐score (DTG‐S) was simply plus or minus the normalized selected DTGs according to their predictive ability for DFS. The DTG‐S classification was the DTG‐S high‐risk group (DTG‐SH) and the DTG‐S low‐risk group (DTG‐SL), separated by the Youden index of the ROC curve.

### 2.4 Construction of nomogram

The results of the multivariate analysis were used to build a nomogram. Calibration curve, c‐index, and ROC curves were used to evaluate the performance of the nomogram for predicting patient DFS.

### 2.5 Propensity score matching

Propensity score (PS) matching was used to eliminate clinical factors that may influence the DFS results. Covariates used for the PS matching included age, ER, PR, T stage, N stage, and tumor grade in the non‐pCR group, with a match capacity equal to 0.05 in SPSS. The associations between the DTG‐S low and DTG‐S high groups and other clinicopathological aspects were analyzed using Student's *t*‐test or Fisher's exact test.

### 2.6 Differential gene expression selection and bioinformatics analysis

Differentially expressed genes (DEGs) in matched groups were selected using the ‘limma’ package in R software with *p*‐values <0.05 and |Log2FC| >1. The bio‐information of DEGs was identified by Gene Ontology (GO) enrichment (biological processes [BP], cellular components [CC], and molecular functions [MF]) and Kyoto Encyclopedia of Genes and Genomes (KEGG) pathway analyses on the DAVID website (https://david.ncifcrf.gov/).

### 2.7 DTG‐S and drug sensitive correlation analysis

The drug_sensitive_AUC data of HER2‐negative breast cancer cell lines and the gene expression data of these cell lines were obtained from the Cancer Therapeutics Response Portal (CTRP) database (http://portals.broadinstitute.org/ctrp/). The relationship of DTG‐S and drug_sensitive_AUC was analyzed by using the Pearson correlation coefficient index in SPSS. Two GEO sets (GSE119262 and GSE150576) were also used to analyze the relationship between the DTG‐S signature and the PI3K pathway inhibitors.

### 2.8 Statistical analysis

The predictive ability and consistency of the DTG‐S classification and the other predictive models for DFS were compared using the ROC and c‐index, respectively. The reclassification improvement of the DTG‐S classification over other prediction models for correcting the risk group of patients was analyzed by the Net Reclassification Improvement (NRI) index and its *p*‐value.^22^

Univariable and multivariable Cox regression analyses were performed to determine whether the prognostic ability of DTG‐S was affected by other factors. Kaplan‐Meier curves and log‐rank tests were generated to illustrate the relationship between the DFS and variables by SPSS (version 25). *p *< 0.05 was considered significantly different in the Cox regression and log‐rank tests.

The relationship between the pCR and DTG‐S classification was analyzed using one‐way ANOVA in SPSS. The predictive ability of DTG‐S for pCR was assessed using ROC curve analysis and a 95% confidence interval (CI). The pCR rate was analyzed between the DTG‐S low (DTG‐SL) and high (DTG‐SH) groups using the chi‐square test in SPSS. Univariate and multivariate logistic regression analyses were implemented and contained DTG‐S and other variables to estimate the odds ratio and its 95% CI for pCR.

As mentioned in the preceding section, R software (version 3.6.1), SPSS (version 25) and Paris 8 were the primary software types used. Statistical significance was defined by a two‐sided *p*‐value <0.05 in all of the analyses.

## 3 RESULTS

### 3.1 Establishing the DTG‐S model

**FIGURE 1:**
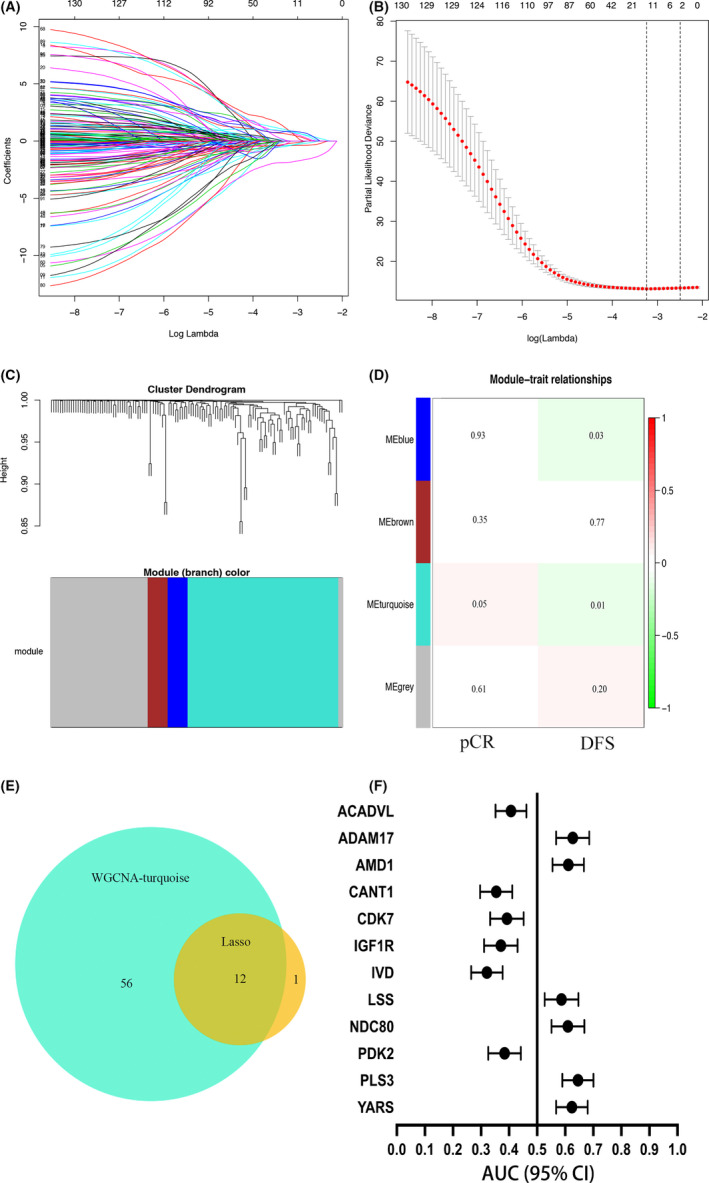
Identification and constitution of DTG‐S. (A) LASSO coefficient profiles. (B) Regression coefficient diagram using LASSO regression analysis. (C) Cluster dendrogram of DTGs. Each branch represents a single gene and each color indicates a single module. (D) Heatmap showing the Pearson correlation between modules and the patients’ pCR and DFS. The numbers in each cell represent the correlation p‐value. (E) Venn diagram representing the overlapping genes between LASSO analysis and the WGCNA turquoise module. (F) The AUC of the ROC curve and its 95% CI for DFS of the 12 selected DTGs in our study

Anthracycline‐ and taxane chemotherapy‐targeted gene identification was obtained from PharmMapper by using the drug's 3D structure, and 132 genes were selected with higher PharmMapper fit scores. Gene expression and clinical information from 466 HER2‐negative breast cancer patients in discovery set GSE25066 were obtained before they received taxane–anthracycline neoadjuvant chemotherapy. To identify the relationship of the 132 DTGs with DFS, we used Lasso logistic regression analysis, identifying 13 possible candidate DTGs associated with patient DFS (Figure 1A,B). The WGCNA divided the 132 DTGs into 4 modules by using average linkage hierarchical clustering (Figure 1C). The module‐clinical correlation analysis shows that the DTGs in the turquoise module were related to both the DFS and pCR of the patients (Figure 1D). After overlapping the 13 DTGs from Lasso analysis and the 68 DTGs from the WGCNA‐turquoise module, we finally selected the 12 overlapping DTGs for further analysis (Figure 1E), and their AUC and 95% CI of the ROC for DFS are shown in Figure 1F. Among the 12 DTGs, high expression levels of ADAM metallopeptidase domain 17 (ADAM17), adenosylmethionine decarboxylase 1 (AMD1), lanosterol synthase (LSS), NDC80 kinetochore complex component (NDC80), plastin 3 (PLS3), and tyrosyl‐tRNA synthetase (YARS) were associated with poor DFS, while low expression level of acyl‐CoA dehydrogenase very long chain (ACADVL), calcium activated nucleotidase 1 (CANT1), cyclin dependent kinase 7 (CDK7), insulin like growth factor 1 receptor (IGF1R), isovaleryl‐CoA dehydrogenase (IVD), and pyruvate dehydrogenase kinase 2 (PDK2) were associated with poor DFS. Based on the expression of the 12 DTGs, we established a simple risk score (DTG‐S) that equals to (ADAM17 expression level)+(AMD1 expression level)+(LSS expression level)+(NDC80 expression level)+(PLS3 expression level)+(YARS expression level)−(ACADVL expression level)−(CANT1 expression level)−(CDK7 expression level)−(IGF1R expression level)−(IVD expression level)−(PDK2 expression level).

### 3.2 DTG‐S with clinicopathological and pCR

**FIGURE 2:**
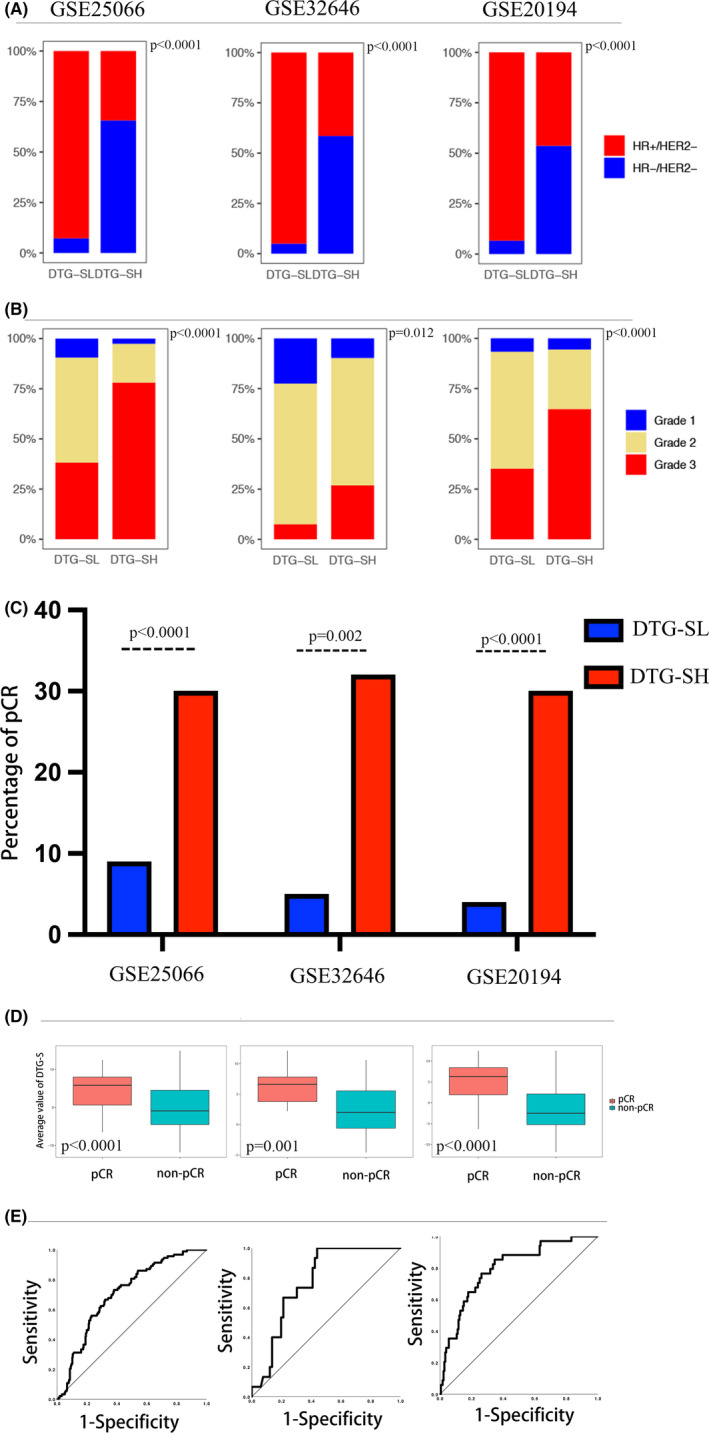
The relationship of DTG‐S with clinicopathological parameters and pCR. Box charts depict the association between the DTG‐S classification and the parameters (molecular types [A] and tumor grade [B]) in each data set. (C) The percentage of pCR rate in the DTG‐SL or DTG‐SH group in each data set. (D) The mean value of DTG‐S in the pCR or non‐pCR group in each data set. (E) The Receiver operating characteristic curve (ROC) analysis of DTG‐S for pCR in each data set. Note: From left to right of each chart, the datasets are GSE25066, GSE32646, GSE20194

We applied the DTG‐S classification to the discovery and verification sets and found that the DTG‐SH group was more likely to be associated with poor clinicopathological features, such as ER negativity, TNM stage III and tumor grade 3 (Figure 2A,B). We also explored correlations of DTG‐S with other published prognostic methods in breast cancer. The DTG‐SH group included more Genomic Grade Index (GGI) and DLDA‐30 high‐risk patients and more Rx Insensitive patients (Figure S1A, C and D). Using the Pam50 intrinsic subtype, we observed that the basal‐like type of breast cancer was increased in the DTG‐SH group, while the Luminal A and Luminal B types were more common in the DTG‐SL group (Figure S1B).

The pCR rate was significantly different between the DTG‐SL and DTG‐SH groups, as patients in the DTG‐SH group exhibited an almost 30% pCR rate, while patients in DTG‐SL presented a pCR rate lower than 10%, which was confirmed in the discovery set GSE25066 and in two verification sets GSE20194 and GSE32646 (Figure 2C). DTG‐S was significantly different in patients with or without pCR, as pCR patients had higher mean DTG‐S values than the non‐pCR patients identified in the discovery and verification sets (Figure 2D). The predictive value of DTG‐S for pCR was confirmed by ROC curve analysis, and the AUC of ROC were 0.732 (95% CI: 0.680–0.783, *p *< 0.0001) in GSE25066 and 0.800 (95% CI: 0.722–0.878, *p*<0.0001) and 0.773 (95% CI: 0.670–0.877, *p *< 0.0001) in GSE20194 and GSE32646, respectively (Figure 1E). As different molecular tumor types have different pCR rates, we analyzed the pCR rate in the HR+/HER2− and HR−/HER2− types independently by DTG‐S classification. The high DTG‐S patients had a higher pCR rate than the low DTG‐S patients in both HR+/HER2− and HR−/HER2− types, as shown in S Figure 1E,F. Using DTG‐S classification and other clinical factors in univariate and multivariate logistic regression analyses revealed that DTG‐S was an independent factor for pCR in both the discovery and verification sets (Table 1). We applied our DTG‐S signature in two independent cohorts in which the NACT regimens did not include anthracycline and taxane to test its regimen specificity. The results showed no significant difference in the ability of DTG‐S to predict a pCR in either cohort (Figure S2A,B). We used several GEO cohorts to explore whether the DTG‐S signature was also useful for pCR prediction in HER2‐positive patients. There were no clear data to show that the DTG‐S signature was useful to predict pCR in HER2‐positive patients (Figure S2C–E).

**TABLE 1:** Univariate and multivariate logistic regression analysis of each variable for pCR

Variables	Univariate logistic regression		Multivariate logistic regression	
GSE25066	OR (95% CI)	*p*	OR (95% CI)	*p*
Age (>50 vs ≤50)	0.782 (0.493–1.239)	0.295	–	–
T (T1&T2 vs T3&T4)	1.067 (0.657–1.732)	0.793	–	–
N (negative vs positive)	0.785 (0.450–1.370)	0.394	–	–
Tumor grade (1&2 vs 3)	0.156 (0.082–0.297)	<0.0001	0.271 (0.133–0.550)	0.027
ER (positive vs negative)	0.215 (0.128–0.359)	<0.0001	0.572 (0.264–1.239)	0.157
PR (positive vs negative)	0.314 (0.186–0.530)	<0.0001	0.979 (0.473–2.026)	0.954
DTG‐S (low vs high)	0.210 (0.121–0.365)	<0.0001	0.463 (0.227–0.943)	0.034
GSE32646				
Age (>50 vs ≤50)	3.30 (0.952–11.435)	0.060	2.583 (0.585–11.409)	0.211
N (negative vs positive)	0.143 (0.018–1.158)	0.068	0.211 (0.023–1.916)	0.167
Tumor grade (1&2 vs 3)	0.207 (0.058–0.737)	0.015	0.570 (0.115–2.820)	0.491
ER (positive vs negative)	0.160 (0.048–0.538)	0.003	0.239 (0.023–2.523)	0.234
PR (positive vs negative)	0.342 (0.099–1.185)	0.091	0.412 (0.338–50.339)	0.267
DTG‐S (low vs high)	0.113 (0.024–0.543)	0.006	0.152 (0.024–0.962)	0.045
GSE20194				
Age (>50 vs ≤50)	1.385 (0.316–6.059)	0.666	–	–
T (T1&T2&T3 vs T4)	0.491 (0.193–1.246)	0.134	–	–
N (negative vs positive)	0.423 (0.151–1.185)	0.102	–	–
Tumor grade (1&2 vs 3)	0.105 (0.035–0.320)	<0.0001	0.258 (0.074–0.902)	0.034
ER (positive vs negative)	0.083 (0.031–0.224)	<0.0001	0.413 (0.103–1.653)	0.211
PR (positive vs negative)	0.168 (0.060–0.468)	0.001	0.547 (0.156–1.921)	0.347
DTG‐S (low vs high)	0.098 (0.035–0.276)	<0.0001	0.267 (0.074–0.957)	0.043

Note

The variables with *p *< 0.1 in univariate logistic regression analysis were selected for the further multivariate logistic regression analysis.

Abbreviation: pCR, pathological complete response; ER, estrogen receptor; PR: progesterone receptor; OR, odds ratio; CI, confidence interval; DTG‐S, drug target gene score; vs, versus.

### 3.3 DTG‐S and DFS

**FIGURE 3:**
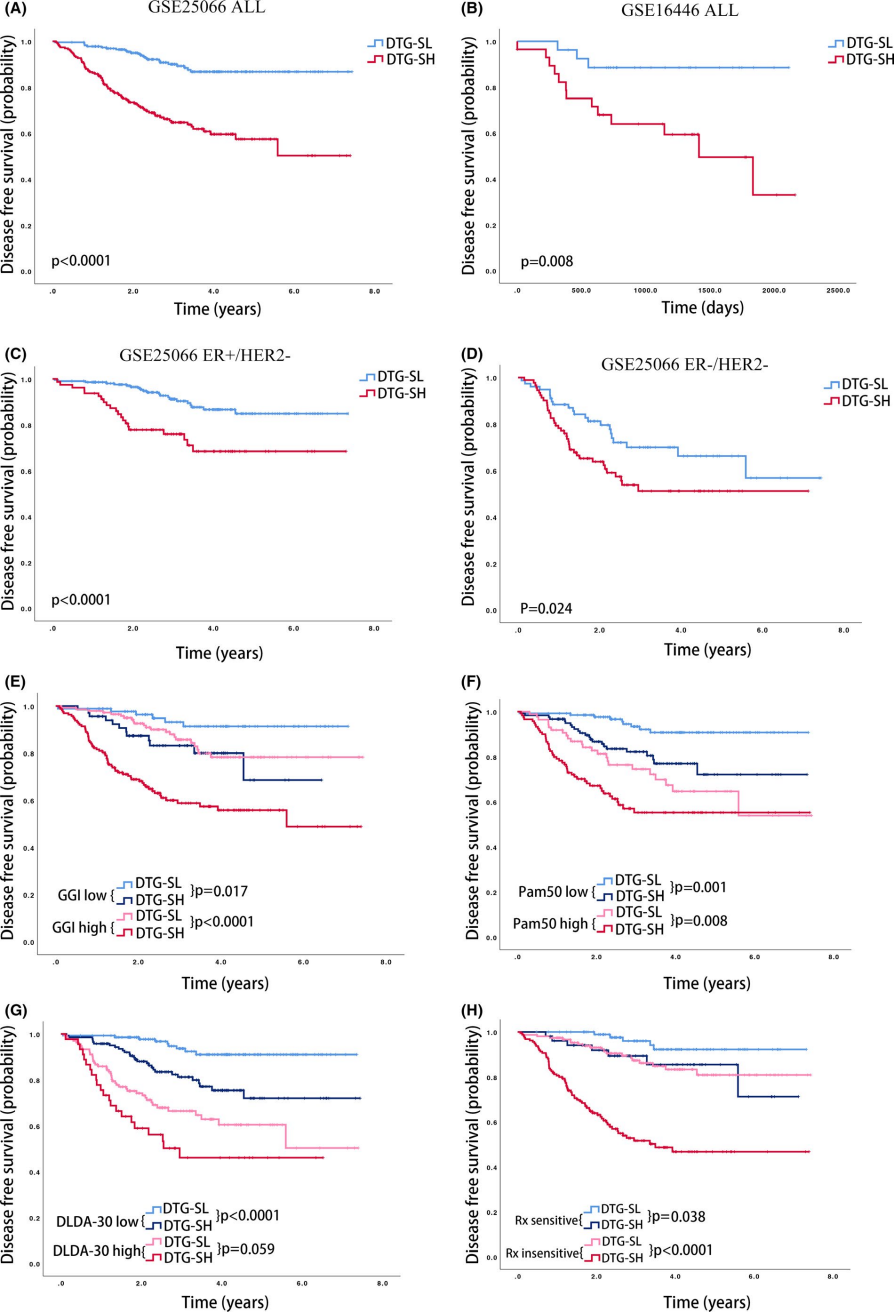
Survival analysis of the DTG‐S classification. Kaplan–Meier curves in all patients according to the DTG‐S classification in GSE25066 (A) and GSE16446 (B). Kaplan–Meier curves in the HR+/HER2‐ subtype (C) and HR‐/HER2‐ subtype (D) according to the DTG‐S classification in GSE25066. Reclassification survival curves according to the DTG‐S classification within the GGI low or high group (E); Pam50 intrinsic Luminal/normal‐like or other subtypes (F); DLDA‐30 low or high group (G); and Rx sensitive or non‐sensitive group (H). *p*‐values were from the log‐rank test analysis

Kaplan–Meier (KM) curves were used to explore the potential role of DTG‐S classification in DFS. We found that DTG‐SH had a worse DFS than the DTG‐SL group in the discovery set GSE25066 and the verification set GSE16446 (Figure 3A,B). We repeated the analysis in each molecular subtype in the discovery set GSE25066 and found the same results for the HR+/HER2− (Figure 3C) and HR−/HER2− subtypes (Figure 3D). The DTG‐S low and high groups showed no difference in DFS for the HER2‐positive patients, as shown in Figure S2F,G.

To investigate whether the DTG‐S classification could reclassify other prognostic models into different risk groups, we applied the DTG‐S classification to the GGI, Rx index, Pam50 intrinsic subtypes, and the DLDA‐30 index. In the GGI signature, the DTG‐S signature reclassified the GGI high‐risk group patients into two significantly different DFS groups, as well as in the GGI low‐risk group (Figure 3E). Similar results were also found for the Pam50 intrinsic subtypes when the patients were classified into Luminal/normal‐like groups and the others (Figure 3F), as well as in the DLDA‐30 index (Figure 3G) and Rx index (Figure 3H). The ROC and c‐index of these predictive models are shown in Table 2, indicating that DTG‐S has a slightly higher predictive ability and consistency than the other models. The positive results of NRI showed that using the DTG‐S in other models improves the risk classification accuracy by 16.9% in GGI, 10.8% in Rx index, 6.5% in Pam50 intrinsic subtypes, and 5.3% in the DLDA‐30 index (Table 2).

**TABLE 2:** Performance of genomic predictors for predicting DFS and the DTG‐S reclassification improvement analysis.

Predictors	AUC (95% CI)	c‐index (95% CI)	NRI (DTG‐S in others)	NRI p value
DTG‐S	0.690 (0.635–0.745)	0.707 (0.619–0.794)	–	–
GGI	0.605 (0.547–0.664)	0.599 (0.511–0.686)	0.169	<0.01
Rx index	0.636 (0.580–0.692)	0.637 (0.549–0.725)	0.108	<0.05
Pam50 intrinsic subtypes	0.608 (0.551–0.664)	0.626 (0.539–0.712)	0.065	>0.05
DLDA‐30	0.663 (0.603–0.724)	0.675 (0.588–0.762)	0.053	>0.05

Note

Performance of the predictive test was biased on the discovery set. The *p* value of NRI were from one‐tailed test.

Abbreviation: DFS, disease free survival; AUC, area under the ROC; CI, confidence interval; DTG‐S, drug target gene score; GGI, gene expression grade index; NRI, net reclassification improvement; DLDA‐30, diagonal linear discriminant analysis‐30 genes.

Univariate and multivariate Cox analyses, including DTG‐S classification, substantial tumor clinicopathological features, or other prognostic values, such as residual cancer burden (RCB), GGI, Rx index and Pam50 intrinsic subtypes, and DLDA‐30 index, showed that DTG‐S was an independent prognostic factor for DFS (HR = 0.216, 95% CI = 0.106–0.439; *p *< 0.0001) (Table 3).

**TABLE 3:** Univariate and multivariate cox regression analysis of variables with DFS

Variables	Univariate analysis		Multivariate analysis	
	HR (95%CI)	*p*	HR (95%CI)	*p*
Age (≤50 vs >50)	0.918 (0.624–1.352)	0.666	–	–
ER (positive vs negative)	0.339 (0.228–0.505)	<0.0001	0.657 (0.338–1.279)	0.216
PR (positive vs negative)	0.370 (0.243–0.564)	<0.0001	0.931 (0.502–1.725)	0.819
T	–	<0.0001	–	0.149
T4	1 (reference)	–	1 (reference)	–
T1	0.220 (0.067–0.722)	–	0.614 (0.172–2.190)	–
T2	0.341 (0.212–0.548)	–	0.593 (0.353–0.998)	–
T3	0.491 (0.296–0.817)	–	0.545 (0.311–0.956)	–
N (N0 and N1 vs N2 and N3)	0.384 (0.256–0.575)	<0.0001	0.646 (0.404–1.032)	0.068
Tumor grade	–	0.026	–	0.453
Grade 3	1 (reference)	–	1 (reference)	–
Grade 1	0.117 (0.016–0.853)	–	0.353 (0.045–2.777)	–
Grade 2	0.681 (0.448–1.035)	–	1.160 (0.710–1.896)	–
pCR vs Non‐pCR	0.277 (0.099–0.517)	<0.0001	0.350 (0.137–0.897)	0.029
DTG‐S (low vs high)	0.199 (0.123–0.321)	<0.0001	0.216 (0.106–0.439)	<0.0001
Rx index (sensitive vs insensitive)	0.220 (0.121–0.403)	<0.0001	0.523 (0.271–1.006)	0.052
GGI (low vs high)	0.366 (0.220–0.608)	<0.0001	0.674 (0.335–1.358)	0.27
Pam50 intrinsic subtypes	–	<0.0001	–	0.642
Basal‐like	1 (reference)	–	1 (reference)	–
Luminal A	0.242 (0.139–0.421)	–	1.511 (0.510–4.476)	–
Luminal B	0.383 (0.206–0.713)	–	1.376 (0.512–3.694)	–
Normal like	0.234 (0.083–0.644)	–	0.691 (0.205–2.331)	–
HER2	0.889 (0.478–1.654)	–	1.504 (0.760–2.974)	–
DLDA−30 (low vs high)	0.268 (0.179–0.401)	<0.0001	0.733 (0.324–1.660)	0.457
RCB (0 vs others)	0.145 (0.059–0.356)	<0.0001	0.198 (0.070–0.563)	0.002

Note

The variables with *p *< 0.05 in univariate cox regression analysis were selected for the further multivariate cox regression analysis.

Abbreviation: DFS, disease free survival; ER, estrogen receptor; PR: progesterone receptor; HR, hazard ratio; CI, confidence interval; DTG‐S, Drug Target Gene Score; vs, versus; RCB, residual cancer burden; pCR, pathological complete response; GGI, Gene Expression Grade Index; DLDA‐30, diagonal linear discriminant analysis‐30 genes

### 3.4 Improvement of the DTG‐S signature

**FIGURE 4:**
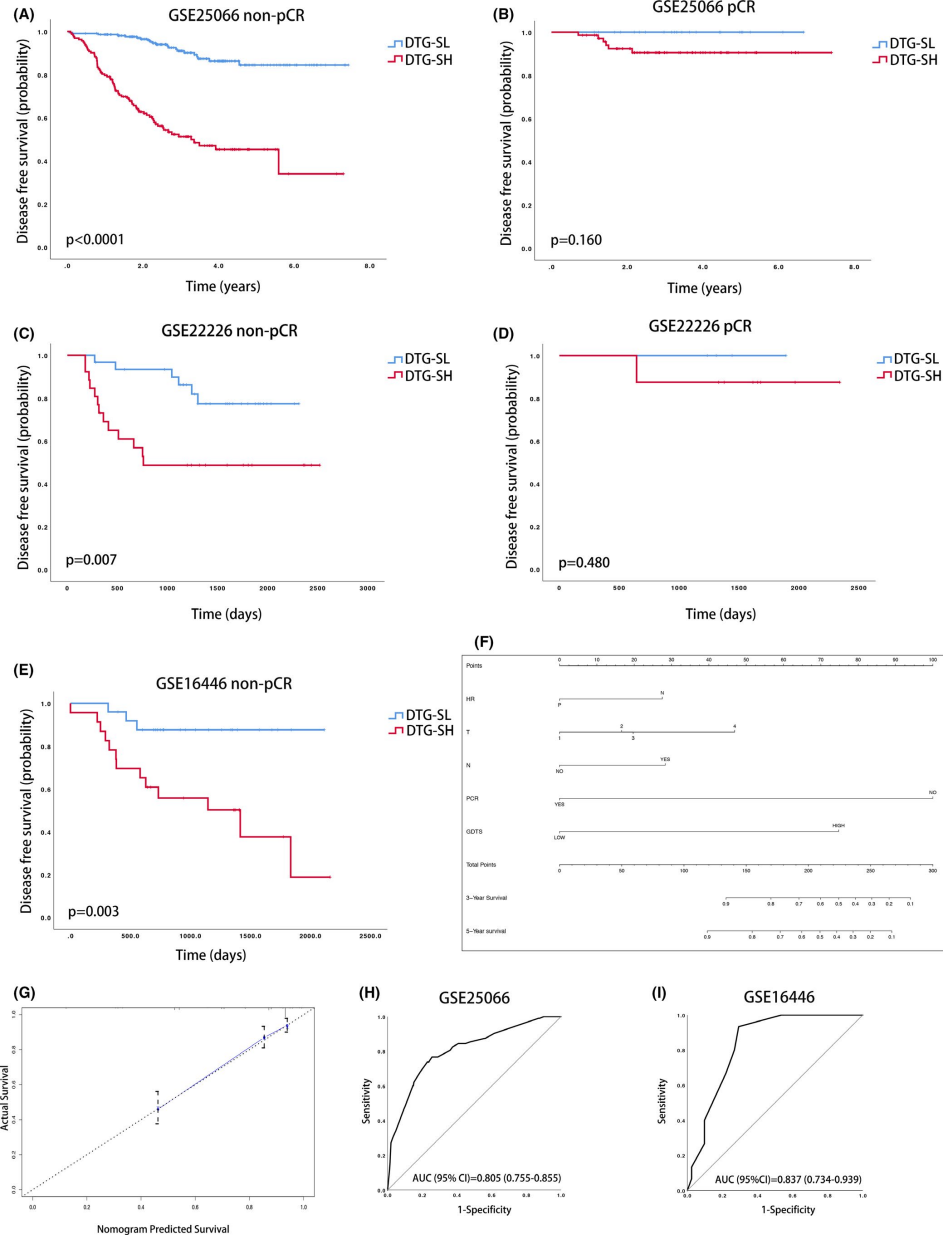
The improvement of DTG‐S classification for DFS. Kaplan–Meier curves in the non‐pCR group (A) and pCR group (B) classified as DTG‐SL or DTG‐SH in GSE25066. Kaplan–Meier curves in the non‐pCR group (C) and pCR group (D) classified as DTG‐SL or DTG‐SH in GSE22226. Kaplan–Meier curves in the non‐pCR group classified as DTG‐SL or DTG‐SH in GSE16446 (E). The nomogram chart for DFS (F). Calibration curve of the nomogram applied in GSE25066 (D). ROC curve of the nomogram for DFS in GSE25066 (H) and GSE16446 (I)

As shown in the multivariate Cox analysis in Table 3 and Table S2, the pCR and DTG‐S classifications were powerful predictors of DFS. To explore the relationship between pCR and DTG‐S, we used KM curves under different conditions. In the non‐pCR group, the DTG‐SL, and DTG‐SH groups showed a significant DFS difference, as patients in the DTG‐SH group had a shorter DFS (Figure 4A [discovery set] and Figure 4C,E [verification set]), while in the pCR group, no significant difference in DFS was found between the DTG‐SL and DTG‐SH patients in the discovery (Figure 3B) and verification set (Figure 4D). As shown in Table S2, DTG‐S, pCR and other clinical variables were associated with DFS.

To improve the DFS prediction ability, we generated a user‐friendly nomogram based on DTG‐S, pCR, hormone receptor, tumor size, and lymph node stage (Figure 4F). The nomogram had higher sensitivity and specificity to predict the DFS for HER2‐negative patients who received NACT. The Calibration curve shows that the nomogram had high consistency between the predicted survival and the real survival (Figure 4G). The c‐index of the nomogram was 0.808 and the area under the ROC for DFS was 0.805 (95% CI: 0.755–0.855) in the discovery set (Figure 4H) and 0.837 (95% CI: 0.734–0.939) in the verification set (Figure 4I).

### 3.5 Possible pathways influencing the DFS

**FIGURE 5:**
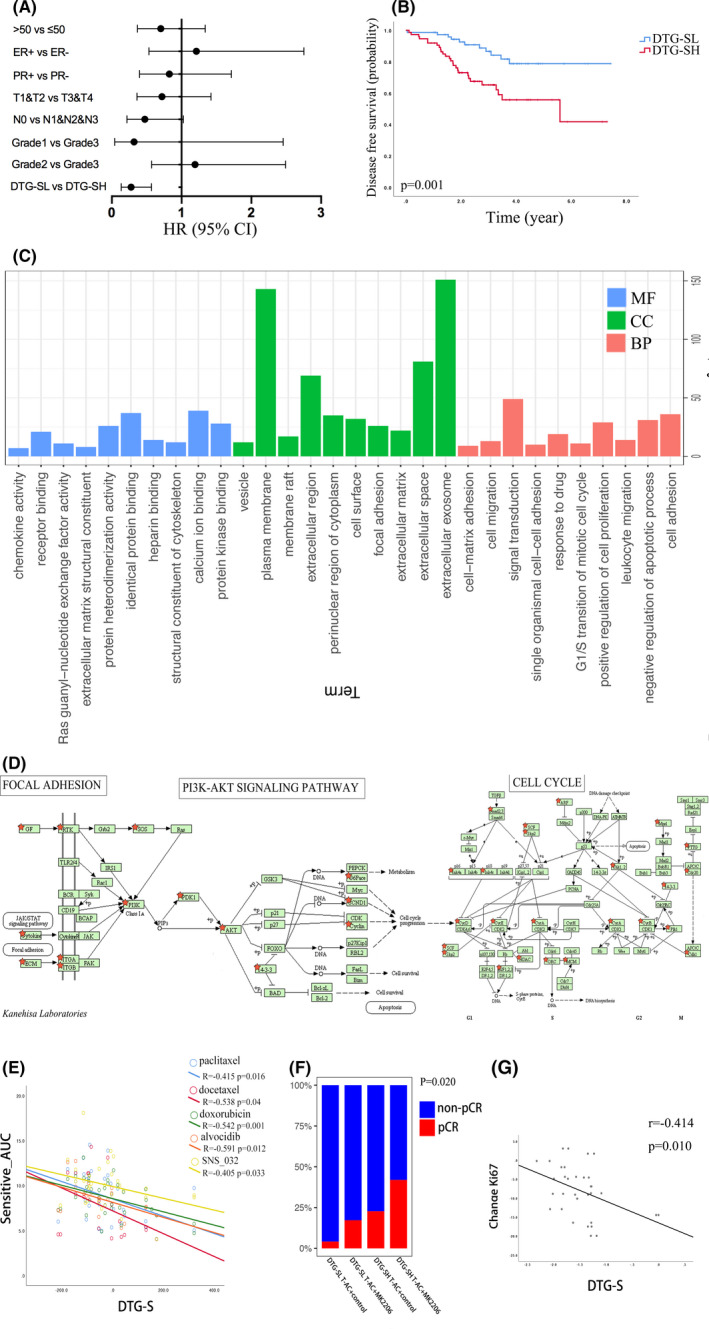
Bioinformatics and drug sensitivity analysis of DTG‐S. (A) Multivariate Cox regression analysis results of each variable in the PS matched patients. (B) Kaplan–Meier curves of DTG‐SL or DTG‐SH for DFS in PS matched patients. Gene ontology (GO) analysis (C) and KEGG pathway analysis (D) of differentially expressed genes (DEGs) obtained from the matched DTG‐SH and DTG‐SL groups. (E) Scatter diagram of drug sensitive AUC and DTG‐S correlation analysis. (F) The percentage of pCR rate in the DTG‐SL or DTG‐SH group treated with MK2206 or not (GSE150576). (G) Scatter diagram of DTG‐S with a change in the Ki67 correlation after neoadjuvant everolimus treatment (GSE119262)

As discussed in the preceding section, high DTG‐S patients exhibited significantly worse DFS in the non‐pCR group. Unmatched variable analysis between DTG‐SL and DTG‐SH patients in the non‐pCR group showed a significant imbalance in most characteristics, such as tumor size, HR status, tumor grade, and molecular characteristics (Table 4). These unbalanced characteristics could affect the DFS, so we used PS matching methods to eliminate these biases to explore the possible mechanism by which they affect the DFS. After PS matching, 74 DTG‐SL, and 74 DTG‐SH patients were selected from the non‐pCR group with no statistically significant differences among all clinical factors (*p *> 0.05, Table 4). Multivariable analysis showed that only DTG‐S was an independent prognostic factor for DFS (Figure 5A) as confirmed in the KM curves. Although all of the other clinical characteristics were not significantly different, DTG‐SH group patients still exhibited a worse DFS than DTG‐SL patients in the non‐pCR group (Figure 5B). Five hundred fifteen differentially expressed genes (DGEs) were identified from the 74 DTG‐SH compared to the 74 DTG‐SL patients. GO analysis of the 515 DEGs showed that these genes were enriched in the cell membrane and extracellular space and influenced the cell cycle, cell migration, signal transduction, and drug response pathways (Figure 5C). KEGG pathway analysis showed that the 515 DEGs were enriched in focal adhesion, cell cycle, pathways in cancer, PI3K‐AKT signaling, and ECM‐receptor interaction pathways (Figure 5D).

**TABLE 4:** Clinicopathological characteristics of DTG‐SL and DTG‐SH in non‐match or PS matched non‐pCR group

Variables	Non‐match cohort			PS match cohort		
	DTG‐SL	DTG‐SH	*p*	DTG‐SL	DTG‐SH	*p*
Age						
≤50	105	84	0.517	40	42	0.434
>50	90	73	–	34	32	–
Tumor size (T)						
T1	12	7	0.003	3	3	0.835
T2	117	68	–	41	37	–
T3	36	53	–	18	23	–
T4	30	29	–	12	11	–
Axillary node status (N)						
N0	72	47	0.103	24	23	0.500
N1&N2&N3	123	110	–	50	51	–
TNM						
I	2	3	0.002	0	0	0.500
II	127	73	–	43	44	–
III	66	81	–	31	30	–
Tumor grade						
1	18	9	<0.0001	5	6	0.948
2	106	49	–	34	33	–
3	71	99	–	35	35	–
ER						
Positive	162	76	<0.0001	54	53	0.500
Negative	33	81	–	20	21	–
PR						
Positive	134	58	<0.0001	38	39	0.500
Negative	61	99	–	36	35	–
Molecular						
HR+/HER2−	169	80	<0.0001	56	53	0.355
HR−/HER2−	26	77	–	18	21	–

Note

Performance of the test was biased on the discovery set.

Abbreviation: pCR, pathological complete response; Num, number; ER, estrogen receptor; PR, progesterone receptor; PS match, Propensity Score Match; DTG‐S, drug target gene score.

### 3.6 Exploring drugs that might be useful for DTG‐SH patients

Next, we searched the CTRP and selected a number of drugs targeted against the identified KEGG pathways. The drug sensitivity and DTG‐S correlation analysis executed in HER2‐negative breast cancer cell lines showed that the higher DTG‐S cells were more sensitive to paclitaxel, docetaxel, and doxorubicin, which is consistent with the preceding clinical analysis results. DTG‐S high scoring cell lines were also sensitive to another two drugs, SNS_032 and alvocidib, which are CDKs inhibitors that influence the cell cycle, suggesting they may be potential means of treatment for DTG‐SH group patients (Figure 5E).

We also explored the association of PI3K‐AKT pathway inhibitors with the DTG‐S signature. In the I‐SPY 2 TRIAL, patients were randomly assigned to receive neoadjuvant therapies, paclitaxel plus MK2206 (an AKT inhibitor), or control drugs, followed by four cycles of AC (doxorubicin and cyclophosphamide). In HER2‐negative patients, high DTG‐S patients had the highest pCR rate in the MK2206 treated group (Figure 5F). Another study treated patients with everolimus (mTOR inhibitor) for neoadjuvant treatment and tested the changes in Ki67 in the patient tissues. We found that the DTG‐S signature was significantly negatively associated with the change in Ki67 which means that high DTG‐S patients may respond well to everolimus treatment (Figure 5G).

## 4 DISCUSSION

In this study, we used anthracycline‐ and/or taxane‐targeted pharmacophores to build a simple score model (DTG‐S) associated with basic clinical features and classical prognostic variables in breast cancer patients who received anthracycline‐and/or taxane‐based neoadjuvant chemotherapy. The DTG‐S classification better classifies patients into different risk groups especially in the non‐pCR group. The nomogram, consisting of the DTG‐S classification, patients’ pathological response condition, hormone receptor conditions, tumor size stage, and lymph node status, could further improve the accuracy of the prediction of DFS. KEGG analysis showed that PI3K‐AKT signaling and the cell cycle pathway affect the malignancy process of DTG‐S high patients, and some PI3K‐AKT and cell cycle inhibitors may be useful for treating these patients.

PharmMapper is a web server and with its large‐scale reverse pharmacophore mapping strategy, it can comprehensively identify potential drug targets.^20^, ^21^ Using the 3D structure of taxane, doxorubicin, and epirubicin, we obtained some pharmacophores, and after the LASSO Cox regression, WGCNA, and ROC prognostic ability analysis, 12 genes were selected for the DTG‐S prognostic model. Most of the 12 genes showed relationships with cancers in different processes. NDC80, CANT1, and IGF1R are involved in cells proliferation, apoptosis, and the cell cycle, which regulates cell growth, differentiation and oncogenic transformation.^23^, ^24^, ^25^, ^26^, ^27^ Some of these genes were previously shown to be prognostic biomarkers in different tumors, for example, high CANT1 expression was related to a poor prognosis in prostate and lung carcinomas,^26^, ^28^ and PLS3 may be useful as a biomarker for identifying recurrences or a poor prognosis in some cancers.^29^, ^30^

Other studies have explored the relationship between these genes and cancer drugs. For instance, PLS3 can enhance p38 MAPK‐mediated apoptosis induced by paclitaxel,^31^ and PDK2 overexpression is closely linked with cisplatin resistance in lung adenocarcinoma, as well as paclitaxel‐resistant lung cancer cells, as combining paclitaxel with a specific PDK2 inhibitor had a synergistic inhibitory effect on paclitaxel‐resistant lung cancer cells.^32^, ^33^ Therapies targeting these genes are now being used in clinical practice, such as mono‐antibodies against IGF‐1R in solid tumors.^34^ The biological and pharmaceutical profiles of these genes indicate that their combination may be a good method to predict the efficacy of anthracycline‐and/or‐taxane‐based neoadjuvant chemotherapy.

The prognosis of NACT patients is associated with classical biological characteristics, such as tumor subtypes, TNM stage, and tumor grade. With the heterogeneity of tumors and treatment strategy improvements, traditional prognostic methods are no longer sufficient to determine the risk of all patients, and there is a trend toward including different indices for different tumor subtypes. Gene expression models, Pam50 intrinsic subtypes, and GGI were previously propose for determining the prognosis of breast cancer patients.^12^, ^35^ In our study, the DTG‐S high group contained more GGI high‐risk patients and basal‐like patients, indicating that this prognostic model shared some consistency with those models. The Rx sensitivity index was built to analyze the response to chemotherapy and the prognosis of breast cancer patients,^36^ and the DTG‐S low group included more Rx sensitive patients (39.9%) than the DTG‐S high group (25.8%). All of these prognostic methods were significant predictors for DFS by univariate analysis, but when combined with DTG‐S in multivariate Cox analysis, only DTG‐S was an independent predictive factor for DFS in anthracycline‐and/or taxane‐based neoadjuvant chemotherapy, indicating the powerful predictive ability of DTG‐S classification.

In our study, the DTG‐S classification was associated with the pathological response to NACT, with a 40% pCR rate in DTG‐S High versus 10% in DTG‐S low, and the multivariate regression analysis showed that DTG‐S was an, but not the only, independent predictive factor for pCR. It is also essential to understand that the prediction of an excellent pathologic response to neoadjuvant chemotherapy does not necessarily predict a good survival. A meta‐analysis study examining the relationship between pCR and DFS or OS concluded that the use of pCR as a surrogate end point for DFS or OS was not supported.^4^ Many factors also influence the response to chemotherapy response and survival, as patients with TNBC have relatively higher pCR rates, but their survival prognoses are poor, whereas patients with luminal‐like breast cancer show a low pCR rate but they have a good prognosis.^37^ The analysis between DTG‐S classification and variables showed a positive correlation between high DTG‐S and classical negative features of breast cancer, such as ER negative, TNM III, and grade III types. This partially explains why the DTG‐S high group was associated with a higher pCR rate but was still closely correlated with a poor prognosis.

Prognostication for patients treated with NACT is important, especially when considering the use of additional postoperative chemotherapy. Classical pCR or non‐pCR classification is widely used, and patients with pCR in most cases show a good prognosis. However, there are some weaknesses in using pCR to determine the prognosis of NACT patients. The first is that pCR rates are different among different breast cancer types: the pCR rate is less than 10% in HR+/HER2− patients and higher than 30% in HR‐/HER2‐ patients.^38^ The second is that a low pCR rate indicates that a large number of non‐pCR patients are classified into the poor prognosis group and thus they may receive further treatment. The DTG‐S showed significant prognostic ability in patients who were receiving NACT. We demonstrated that patients in the pCR group showed no significant prognostic difference between DTG‐SL and DTG‐SH, while in the non‐pCR group, DTG‐SL and DTG‐SH had significantly different predictions even after balancing the classical clinicopathology prediction factors. The nomogram combined with the DTG‐S signature and some classical predictors of breast cancer can further improve the ability to predict survival. These findings indicate that DTG‐S classification adds some prediction power for patients and it should be used in clinical trials in the future to verify whether it can assist in making decisions about adjuvant therapy for patients.

The survival differences were influenced by many variables, except pCR or non‐pCR, in patients with NACT. Previous studies have proved that ER‐negative breast cancer, especially TNBC and grade 3 tumors, generally has a higher proliferation rate and a more aggressive biological behavior,^39^, ^40^ and these poorly differentiated tumors are more likely to respond to cytotoxic chemotherapy but still have a poor prognosis.^41^ KEGG and GO analysis of the balanced patients revealed some pathways associated with a poor prognosis in the high DTG‐S group, such as cell cycle, cell migration, and cell signal transduction, such as the PI3K‐AKT signaling pathways. The PI3K‐Akt pathway is involved in different processes in breast cancer, such as cellular proliferation, differentiation, migration, apoptosis, and chemoresistance, and is highly active in breast cancer cells.^42^ As reported, the PI3K‐AKT pathway plays a crucial role in the cell cycle regulatory machinery, and all of these active pathways contribute to a poor prognosis in breast cancer.^43^ Therefore, blocking PI3K‐AKT and the cell cycle pathway may be helpful for DTG‐SH breast cancer patients. We subsequently analyzed some drugs targeting the PI3K‐AKT and cell cycle pathways, and the results were consistent with the pathway analyses. According to our analysis, treatment targeting these pathways might have a degree of superiority in DTG‐S high patients. We think our results could help with drug selection in future neoadjuvant clinical trials.

Although the DTG‐S signature showed excellent predictive ability in HER2 negative breast cancer patients, some limitations of our study should be taken into consideration. First, our study was based on several GEO datasets, which were retrospective and may have had some selection bias. We need more independent cohorts, especially prospective cohorts, to validate our DTG‐S signature and prognostic nomogram. Second, we excluded HER2‐positive patients in our study because HER2‐targeted therapy is very important for HER2‐positive breast cancer and may influence the predictive ability of the DTG‐S signature. Therefore, in the future, we should validate our signature in different molecular types of breast cancer. Third, although our DTG‐S signature was derived from anthracycline and taxane target pharmacophores, we still need additional data to demonstrate its specificity, as some patients are not treated with anthracycline +/− taxane. Finally, we need further experiments to clarify the underlying mechanism of our DTG‐S signature in order to find more therapies for high‐risk patients.

In summary, our 12‐gene DTG‐S signature displays excellent predictive values for DFS and for a pathological response to anthracycline‐and/or taxane‐based chemotherapy in HER2‐negative breast cancer. DTG‐S classifies patients into very accurate risk groups, especially patients without pCR after NACT, and it adds significant prognostic and predictive value to classic prognostic factors. The biological relevance of our signature is its involvement in cell cycle, cell migration, and cell signal transduction. Targeted drug analysis showed that some CDK inhibitors that block the cell cycle and PI3K‐AKT pathway inhibitors may be a useful treatment for high DTG‐S patients. Our signature contains a small number of genes and it can be easily be applied in the clinic. In the future, large‐scale prospective studies are needed to validate these results.

## Conflicts of interests

The authors declare that they have no competing interests.

## Author contributions

XL and HFS contributed to the study design. XL and QQL contributed to data collection. XL, QQL and YL performed statistical analysis, interpretation, and drafted the manuscript. YFH, WJ and HFS revised the manuscript. All authors contributed to critical revision of the final manuscript and approved the final version of the manuscript. XL, WJ, YFH, and HFS provided financial support and study supervision.

## Ethics approval

No ethics approval was required for this work. All data in this study are publicly available.

## Supporting information

Figure S1

Figure S2

Table S1

Table S2

## Data Availability

The datasets used and analyzed during the current study are available from the Gene Expression Omnibus (GEO) (https://www.ncbi.nlm.nih.gov/geo/), PubChem (https://pubchem.ncbi.nlm.nih.gov/), PharmMapper (http://www.lilab‐ecust.cn/pharmmapper/), CTRP (https://portals.broadinstitute.org/ctrp.v2.2/) and DAVID (https://david.ncifcrf.gov/).
