# Supplementary figures and images for: A pharmacophore‐based classification better predicts the outcomes of HER2‐negative breast cancer patients receiving the anthracycline‐ and/or taxane‐based neoadjuvant chemotherapy
Source: Cancer Med. 2021 Jun 2;10(13):4658–74. doi: 10.1002/cam4.4022 (PMC8267145; doi:10.1002/cam4.4022)

**A**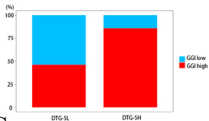**B**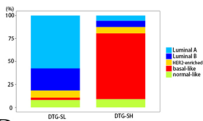**C**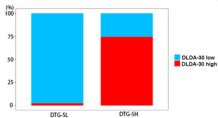**D**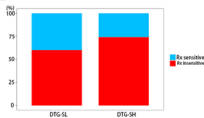**E**

HR+/HER2-

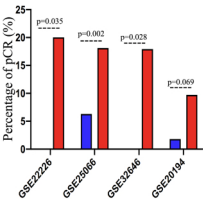**F**

HR-/HER2-

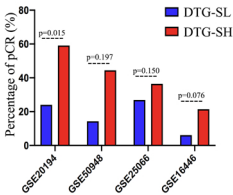

Supplement: Supplementary file 1 — Figure S1 [file CAM4-10-4658-s002.pdf]

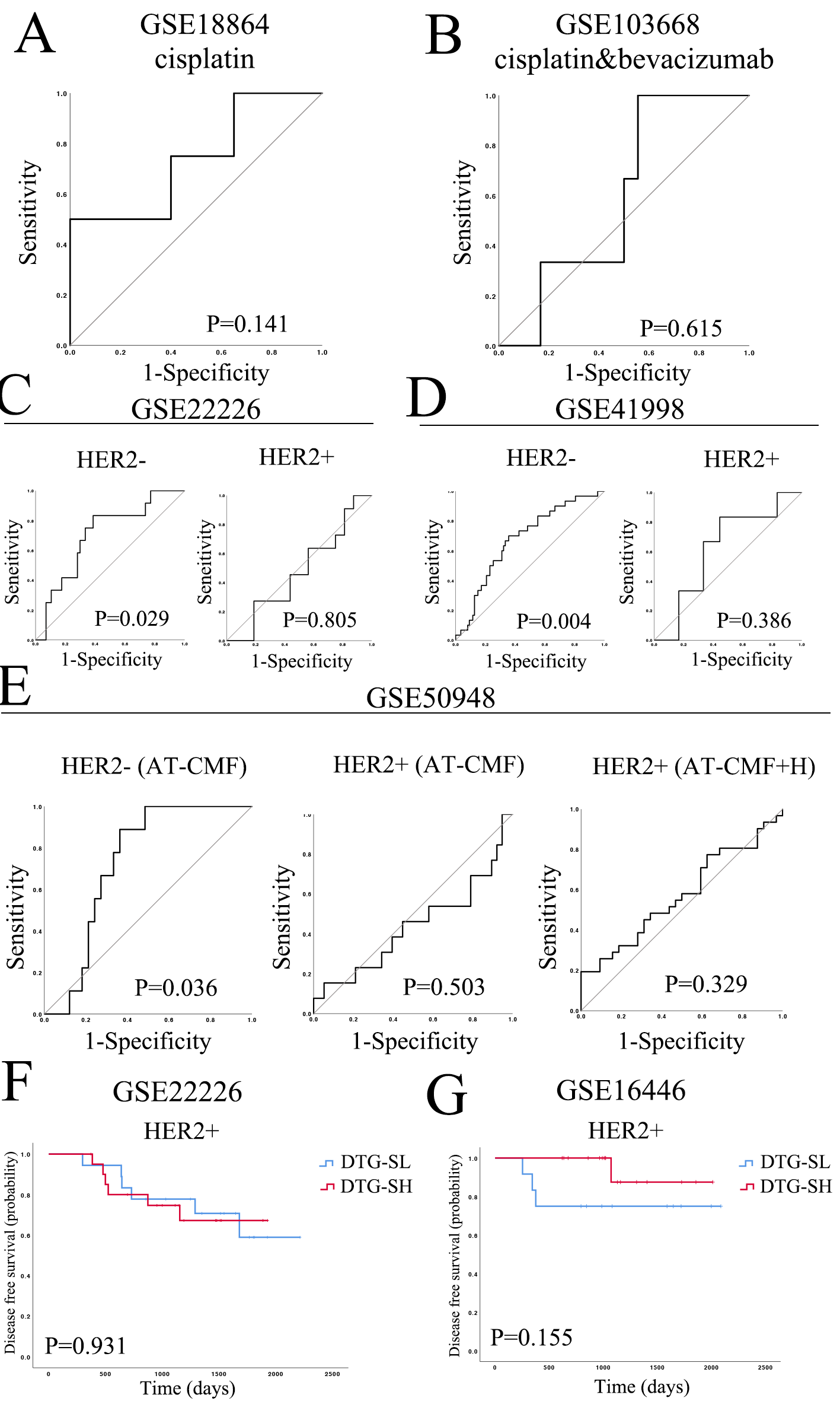

Supplement: Supplementary file 2 — Figure S2 [file CAM4-10-4658-s004.tif]
